# Mean Urea Reduction Ratio among Patients Undergoing Hemodialysis at a Tertiary Care Centre: A Descriptive Cross-sectional Study

**DOI:** 10.31729/jnma.8166

**Published:** 2023-05-31

**Authors:** Krishna Kumar Agrawaal, Sanjog Kandel, Sudhan Devkota

**Affiliations:** 1 Nephrology Unit, Department of Internal Medicine, Universal College of Medical Sciences, Bhairahawa, Rupandehi, Nepal; 2 Department of Internal Medicine, Universal College of Medical Sciences, Bhairahawa, Rupandehi, Nepal

**Keywords:** *chronic kidney disease*, *dialysis*, *hemodialysis*

## Abstract

**Introduction:**

Chronic kidney disease is a major cause of mortality with a prevalence of 6%. Over the past half-century, hemodialysis has been the most preferred modality of treatment for sustaining the life of patients with end-stage kidney disease. Despite hemodialysis being freely available, achieving adequacy in hemodialysis is a challenging task. Inadequate dialysis is responsible for the high mortality. This study aimed to find out the mean value of the urea reduction ratio among patients undergoing hemodialysis at a tertiary care centre.

**Methods:**

This was a descriptive cross-sectional study conducted from 15 January 2023 to 15 April 2023. Ethical approval was taken from Institutional Review Committee (Reference number: UCMS/IRC/044/23). Patients aged >18 years, undergoing maintenance hemodialysis and giving informed and written consent were included in the study. Urea reduction rate and single-pool Kt/V were estimated. Convenience sampling method was used.

**Results:**

Among 100 patients, the mean urea reduction ratio among the study population was 25.24±15.59%. Males represented 62 (62%) of the study population. The mean age was 47.9±14.74 years. Hypertension and Diabetes mellitus was found to be the leading cause of end-stage kidney disease with 61 (61%) and 27 (27%) respectively. The mean value of spKT/V was 0.73±0.162.

**Conclusions:**

The mean urea reduction ratio was found to be lower than the other studies done in similar settings.

## INTRODUCTION

Chronic kidney disease (CKD) is an emerging disease with a prevalence of 6% in Nepal.^1^ Over the past halfcentury, there has been a remarkable achievement in hemodialysis (HD) as a modality for renal replacement therapy.^2^ Beginning from the initiation of hemodialysis at Bir Hospital in 1987,^3^ Government of Nepal has been providing free hemodialysis since 2016.^4^ According to Renal Physicians Association and the National Kidney Foundation's disease outcomes quality initiative, dialysis adequacy is estimated by calculating urea reduction ratio (URR) and/or KT/V where (K: clearance of urea, T: duration of dialysis, V: distribution of urea).^5^

Due to logistics and financial constraints, most hemodialysis centres are providing twice-a-week hemodialysis and reusing dialysers which may affect the quality of hemodialysis.^6,7^ It is imperative to know the quality of HD provided at individual centres. Hence, we designed this study to estimate URR and single pool KT/V in patients undergoing HD.

This study aimed to find out the mean value of the urea reduction ratio among patients undergoing hemodialysis at a tertiary care centre.

## METHODS

This was a descriptive cross-sectional study conducted from 15 January 2023 to 15 April 2023 under the Nephrology unit, Department of Internal Medicine at Universal College of Medical Sciences, Bhairahawa, Rupandehi, Nepal. Ethical approval was taken from the Institutional Review Committee of the same institute (Reference number: UCMS/IRC/044/23). Patients aged >18 years, undergoing maintenance hemodialysis and giving informed and written consent were enrolled in the study. Those who did not give informed consent were excluded. Convenience sampling was done and the sample size was calculated using the formula:


$$\begin{array}{ll} \text{n} & =\frac{{\text{Z}}^{2}\times \sigma^{2}}{{\text{e}}^{\text{2}}}\\ & =\frac{1.96^{2}\times 21.48^{2}}{5^{2}}\\ & =71 \end{array}$$

Where,

n = minimum required sample size
Z = 1.96 at 95% Confidence interval (CI)
σ = standard deviation is taken as 21.48 from published literature^8^
q = 1-p
e = margin of error, 5%

Here, the minimum required sample size was 71. However, 100 sample size was taken. The measurement of blood urea before and after dialysis was done and the calculation of URR and spKT/V was done using the formulas mentioned below:

**Urea Reduction Ratio (URR)**


$$\text{URR=}\frac{\text{(pre-dialysis Urea - post-dialysis Urea)}}{\text{(pre-dialysis Urea) }\times \text{100\%}}$$

**Single-Pool Index (spKt/V)**

Parameters spKt/V and URR are connected mathematically as follows:

SpKt/V = - *In* (1 - URR),

Where *In* stands for natural logarithm.

Several studies have shown that if the rate of KT/V reaches 1.2 or URR is more than 65%, this is effective in improving dialysis patients' prognosis.^5^

The available demographic, clinical and laboratory parameters were recorded as per the proforma. Data were entered and analyzed using IBM SPSS Statistics version 17.0.

## RESULTS

A total of 100 participants were enrolled in the study. About 62 (62%) were males (Table 1).

**Table 1:** Baseline characteristics (n = 100).

Characteristics	Categories	n (%)
Age (years)	18-39	30 (30)
	40-59	47 (47)
	≥ 60	23 (23)
Gender	Male	62 (62)
	Female	38 (38)
Religion	Hindu	90 (90)
	Muslim	9 (9)
	Buddhist	1 (1)
Education	Illiterate	27 (27)
	Up to secondary education	60 (60)
	Above secondary education	13 (13)
Hemodialysis frequency	Once a week	4 (4)
	Twice a week	62 (62)
	Three times a week	34 (34)
Type of Vascular access	Radiocephalic arteriovenous fistula	50 (50)
	Brachiocephalic arteriovenous fistula	29 (29)
	Internal Jugular Catheter	21 (21)

The mean URR among the study population was 25.24±15.59% whereas spKT/V was 0.73±0.162 (Table 2).

**Table 2:** Mean URR and spKT/V based on gender (n = 100).

Characteristics	spKT/V Mean±SD (%)	Urea Reduction Ratio Mean±SD (%)
Male	0.739±0.161	24.76±15.08
Female	0.735±0.166	26.02±16.5)

**Figure 1:**
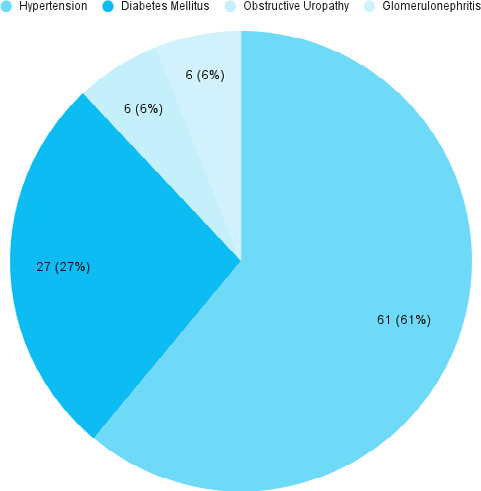
Underlying conditions (n= 100).

Hypertension was present in 61 (61%) patient's requiring hemodialysis (Figure 1).

The mean Hemoglobin level is 8.11±1.83 gm/dl (Table 3).

**Table 3:** Laboratory parameters (n= 100).

Laboratory parameters	Mean±SD
Hemoglobin (gm/dl)	8.11±1.83
Serum Calcium (mg/dl)	8.06±0.43
Serum Phosphorous (mg/dl)	7.39±2.50
Serum albumin (gm/dl)	3.21±0.45
Iron Saturation (%)	28.48±3.45
Pre-HD Serum Urea (mg/dl)	176.12±59.70
Post HD Serum Urea (mg/dl)	130.88±52.19
Pre HD serum Creatinine	10.70±4.26
Post-HD Serum Creatinine	9.52±4.02

Mean URR among patient undergoing hemodialysis thrice a week is 23.07±15.05 and mean spKT/V was 0.75±0.17 (Table 4).

**Table 4:** Mean URR and spKT/V according to the number of HD sessions per week (n= 100).

Characteristics	Once a week (n = 4)	Twice a week (n = 62)	Thrice a week (n = 34)
Urea reduction ratio (%)	26.72±17.20	26.33±15.92	23.07±15.05
Single pool spKt/V	0.72±0.17	0.73±0.15	0.75±0.17

Vascular accesses used for hemodialysis were arteriovenous fistula which included Radiocephalic arteriovenous fistula (RCAVF) 50 (50%), brachiocephalic arteriovenous fistula (BCAVF) 29 (29%) and temporary internal jugular venous catheter (IJV) 21 (21%) (Table 5).

**Table 5:** Mean URR and spKT/V as per vascular access for HD (n= 100).

Type of vascular access	spKT/V Mean±SD (%)	Urea Reduction Ratio Mean±SD (%)
RC AVF	0.71±0.16	27.30±15.72
BC AVF	0.78±0.15	19.70±13.52
IJ Catheter	0.71±0.12	27.97±16.70

## DISCUSSION

This study highlighted the importance of estimation of URR and calculating spKT/V in patients undergoing hemodialysis. In our study of 100 hemodialysis patients, males were more in number 62 (62%) than females 38 (38%), which was similar to a study done in Chitwan, Nepal.^9^ The mean age of patients in our study was 47.9±14.7 years which was similar to other studies.^10^ The commonest cause of end-stage kidney disease (ESKD) in our study was hypertension 61 (61%) followed by type 2 diabetes mellitus 27 (27%) and chronic glomerulonephritis 6 (6%) whereas in another study it was found that diabetic nephropathy followed by hypertension were common causes of ESKD.^11,12^ On the contrary, another study found chronic glomerulonephritis to be the most common cause followed by diabetic nephropathy.^13^ Mean Hemoglobin level in our study population was found to be 8.11±1.83 gm/dl which was similar to the study done at a centre in Kathmandu.^14^ The mean calcium level in the study population was found to be 8.06±0.43 mg/dl which is similar to findings in a retrospective study conducted at KIST Medical College Teaching Hospital.^15^ Similar results were also observed in a study from Bir Hospital.^16^ Hypocalcaemia is a part of abnormal mineral metabolism in CKD and evident from stage 4 CKD.^17^ Analysis of our data showed mean pre-dialysis urea was 176.12±59.70 mg/dl and mean post-dialysis urea was 130.88±52.19 mg/dl. Reanalysis of the primary data from the national cooperative dialysis study showed that Kt/V <0.8 was associated with a relatively high rate of patient morbidity, whereas Kt/V values between 1.0 and 1.2 were associated with better outcomes.^18^

Mean URR in our study was 25.24±15.59 % and Mean Kt/v as assessed by Jindal's equation was 0.73±0.162. This was lower than a study from Nepal where the mean URR was 65.3% and mean Kt/v was 0.99.^19^ Similar discrepancy was also seen in a study conducted at the Institute of Medicine, Maharajgunj where the mean spKt/V and URR were 1.15±0.3 and 60.35±9.26% respectively.^20^ Various factors are accountable for lower URR and spKt/V. The average time of dialysis treatment received by patients could be actually less than the prescribed treatment time. The length of each hemodialysis session and low-flux membranes used for HD could be a justifiable reason for such discrepancies in various studies. Patients could not reach the dialysis unit at the scheduled time or had to leave before the completion of the prescribed time due to transportation and scheduled timing issues. Hence considering these factors for lower values of URR and spKt/V, it is necessary to take measures to improve the quality of dialysis.

Limitations such as confounding factors for Kt/V were not collected in this study which includes albumin, ferritin, residual renal function, and other comorbidities of the study population.

## CONCLUSIONS

The mean urea reduction ratio was found to be lower than the other studies done in similar settings.
